# Induced allopatry as main mechanism explaining trap catch reduction in low dose mating disruption trials on the strawberry pest *Acleris comariana* (Lepidoptera: Tortricidae)

**DOI:** 10.1002/ps.8877

**Published:** 2025-05-09

**Authors:** Glenn Peter Svensson, Victoria Tönnberg, Linda‐Marie Rännbäck, Fredrik Andersson, Erik Hedenström, Lene Sigsgaard

**Affiliations:** ^1^ Department of Biology Lund University Lund Sweden; ^2^ HIR Skåne Borgeby Sweden; ^3^ Eco‐Chemistry, Department of Natural Sciences, Design and Sustainable Development Mid Sweden University Sundsvall Sweden; ^4^ Department of Plant and Environmental Sciences University of Copenhagen Copenhagen Denmark; ^5^ Department of Plant Sciences Norwegian University of Life Sciences Ås Norway

**Keywords:** mating disruption, *Acleris comariana*, sex pheromone, aggregation, dispenser, integrated pest management

## Abstract

**BACKGROUND:**

The strawberry tortrix, *Acleris comariana* (Lepidoptera: Tortricidae), is a destructive pest of strawberry in Denmark and southern Sweden. The efficacy of pheromone‐based communication disruption of the species was examined in crop fields in southern Sweden. Due to the high cost of purchasing or synthesizing the pheromone (*E*)‐11,13‐tetradecadienal, lower quantities were applied per ha compared to similar mating disruption studies on other tortricid pests.

**RESULTS:**

When treating 1 ha within fields with 14 or 1.4 g of pheromone and using rubber septa as dispensers, trap catches were reduced by ≥98% *versus* control areas. When treating whole fields with 0.45–0.90 g/ha and using 1 g SPLAT droplets as dispensers, the effect on trap catch was less pronounced (63–95% reduction *vs* control fields). A corresponding reduction in larval numbers following the treatment was not achieved. Additional experiments revealed that males are more attracted to SPLAT droplets compared to trap lures, and aggregate near SPLAT droplets, indicating that low catches in traps were due to induced allopatry, a form of competitive disruption. In addition, female‐baited traps were outcompeted when placed close to septum‐baited traps. Pest densities were high, and the lack of control effect could be attributed to high encounter rates between the sexes despite the female competitive disadvantage, making mating disruption less efficient.

**CONCLUSION:**

Our data show the potential for pheromone‐based control of *A. comariana* as part of integrated pest management, but the method needs optimization regarding density and strength of dispensers and ways to reduce the initial density of the pest to levels where competitive mechanisms of mating disruption can be efficient. © 2025 The Author(s). *Pest Management Science* published by John Wiley & Sons Ltd on behalf of Society of Chemical Industry.

## Graphical abstract

This study evaluated pheromone‐based mating disruption of the strawberry pest *Acleris comariana* (Lepidoptera: Tortricidae). In treated crop areas males were more attracted to the dispensers than to the trap lures and aggregated near such dispensers, explaining the low catches in traps in these areas as an effect of competitive disruption *via* induced allopatry. With high densities of the pest in the experimental fields, the pheromone treatment was not sufficient to reduce the encounter rates between sexes, and thus the mating frequency and number of offspring larvae in the subsequent generation. Optimization of the method is thus needed for successful mating disruption of *A. comariana*.

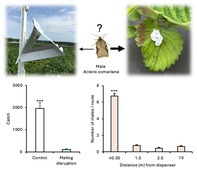

## 1 INTRODUCTION

Insect sex pheromones have been used for early detection, monitoring and control of agricultural pests for several decades.^1^ With more restrictions on traditional insecticides, the global market for pheromones is growing rapidly, and has been estimated to increase from 4.4 billion USD in 2023 to 13.3 billion USD in 2030.^2^ Pheromone‐mediated mating disruption offers a sustainable alternative targeting the mobile adult stage of the pest by releasing high doses of synthetic pheromone in the crop field to disrupt the communication between the sexes and prevent or delay mating. This is a particularly suitable control method for species with a hidden lifestyle, which are difficult to control using traditional insecticides. Mating disruption is increasingly and successfully applied to control major lepidopteran pests in horticulture, including the tortricids *Cydia pomonella* (L.), *Grapholita molesta* (Busck) and *Lobesia botrana* Denis & Schiffermüller.^3^

The strawberry tortrix, *Acleris comariana* Lienig and Zeller (Lepidoptera: Tortricidae), has become a major pest of strawberries in Denmark and southern Sweden.^4^, ^5^, ^6^ The biology of this moth is well described.^7^, ^8^ The first flight occurs from June to July, and the second flight occurs from September to November. Eggs laid in the autumn hibernate and hatch in the spring coinciding with flowering of the crop. The larvae feed on leaves and flowers, and flower‐feeding results in small and asymmetric berries or abortion of berries. The second generation of larvae only feeds on leaves, but the number of overwintering eggs from that generation will determine the pest pressure on the berry crop the next season. Observation of small and malformed berries to estimate *A. comariana* infestation levels is not reliable because other factors can cause the same modified morphology of berries, for example, poor pollination,^9^, ^10^ transfer of pollen of low quality,^11^ frost,^12^ or infestation by other tortricid moths or plant bugs.^13^, ^14^ Instead, survey of larvae can be a better predictor of future infestation levels of the pest. The available control methods for *A. comariana* are not sufficient to control the pest and alternatives are urgently needed. The female‐produced sex pheromone of *A. comariana* was recently identified as (*E*)‐11,13‐tetradecadienal (*E*11,13‐14:Ald) and found to be very efficient in attracting males to traps in commercial strawberry fields.^15^

Several mechanisms have been suggested to cause mating disruption in moths. Miller and Gut^3^ divided such mechanisms into competitive and non‐competitive disruption. Competitive mechanisms do not impair the ability of males to respond to the sex pheromone, and therefore, males can respond to females and traps and the effect of the pheromone treatment would depend on the ratio between dispensers and conspecific calling females in the field. In contrast, non‐competitive mechanisms reduce the ability of males to detect and respond to the pheromone, and such mechanisms are independent of the ratio between dispensers and calling females.^16^ Analysis of disruption profiles from case studies suggested that competitive disruption is the main mechanism of pheromone‐based population control,^17^ and this hypothesis was supported by large‐cage field experiments on *C. pomonella*.^18^ The main mechanisms causing competitive disruption are (i) *competitive attraction* when males track pheromone plumes from dispensers all the way to the source but do not aggregate near such point sources and (ii) *induced allopatry* when males are attracted to and aggregate near dispensers.^3^ In both scenarios, males may be diverted from females and catches in monitoring traps are drastically reduced in pheromone‐treated fields. The relative density and strength of the different point sources (females, dispensers and traps) will determine the probabilities of males locating calling females *versus* ending up in traps. A strong reduction in trap catch is often not translated into lower level of mated females or fewer offspring larvae and damage in the subsequent generation, because the density of calling females is much higher than those of dispensers and traps.

Reports on the use of *E*11,13‐14:Ald as sex pheromone component in moths have so far been restricted to *Acleris* (Pherobase.com), but the fact that the genus includes several important pests^19^ makes the market for *E*11,13‐14:Ald as disruptant potentially large. No attempts to control an *Acleris* species using pheromone treatment have, however, been made. In this study, we performed field experiments in southern Sweden to evaluate mating disruption as a potential control tactic against *A. comariana* and examined possible mechanisms responsible for the disruption effect. Due to the high cost of both purchasing and synthesizing larger amounts of the compound *E*11,13‐14:Ald, lower quantities of pheromone were applied per ha compared to similar mating disruption studies on other tortricid pests. The effect of the pheromone treatment was evaluated by comparing trap catches of males and frequencies of mated females in treated *versus* untreated areas, and by estimating densities of larvae in the next generation in treated *versus* untreated areas. Additional experiments were performed to better understand the mechanism causing the disruption effect in this study. The hypothesis that competitive disruption was the main mechanism causing reduced trap catches in treated areas was tested by trapping experiments using dispensers as lures and by direct observations of the flight behavior of males near such dispensers. In addition, attraction of males to female‐baited traps *versus* septum‐baited traps was investigated to check how well females compete with monitoring traps, which would aid in understanding the trap catch data collected in this study.

## 2 MATERIALS AND METHODS

### 2.1 Chemicals, baits, dispenser systems and traps

Synthetic *E*11,13‐14:Ald was purchased from Pherobank (Wijk bij Duurstede, the Netherlands) (≈90% chemical purity) or synthesized by us (several batches with 88–99% chemical purity, see Supplementary Information). To produce trap baits and dispensers for the experiments 2020–2021, compound solutions were prepared in *n*‐heptane (>99%) and 100 μL of a solution applied on red rubber septum (11 × 5 mm, #224100–020) from Wheaton Science Products (Millville, NJ, USA). To stabilize the aldehyde the test solutions contained 1% of the antioxidant 3‐tert‐butyl‐4‐hydroxanisole (BHA). Trap baits were loaded with 100 μg of *E*11,13‐14:Ald, whereas septa to be used as dispensers were loaded with 70 mg (2020) or 7 mg (2021) of *E*11,13‐14:Ald. In the experiments 2023–2024, SPLAT (Specialized Pheromone and Lure Application Technology, ISCA, Riverside, CA, USA), a biodegradable wax matrix providing long‐term rain and UV protection for the pheromone, was used as a dispensing system. Neat *E*11,13‐14:Ald was incorporated into the wax at the recommended dose of 1.5% by weight and added to plastic tubes before being shipped. In the experiments 2019, CSalomon delta traps were used (Plant Protection Institute, Hungarian Academy of Science, Budapest, Hungary), and in the remaining experiments, CAPTA or Biobest delta traps were used (Biobasiq, Malmö, Sweden). In all experiments, traps were hung at 1 m height using plastic fence poles.

### 2.2 Rubber septa release kinetic study

The release rate of *E*11,13‐14:Ald from rubber septum dispensers loaded with 7 or 70 mg of the compound was determined gravimetrically. For each dose, two dispensers were hung in a wind tunnel at 23 °C, 50% r.h., and 0.3 m/s wind speed, and every 1–4 days, they were weighed to four decimals places on a Mettler AE 240 balance to determine release rate of the pheromone.

### 2.3 SPLAT release kinetic study

A set of SPLAT droplets was maintained in the field in July–August 2023 and 2024 and individual droplets collected at different times to analyze how much of *E*11,13‐14:Ald that was left in the wax. The weight of a SPLAT dispenser dropped by approximately 30% during the first day of the experiment due to water loss, but then remained the same throughout the period of analysis, which enabled reliable quantification of the pheromone in the droplets. Dispensers were analyzed after 1 day, 1 week, 2 weeks, 4 weeks, and 6 weeks (two droplets per age group). Each 1 g droplet was weighed, placed in a glass vial (Kimax), and extracted in 10 mL of *n*‐heptane in a water bath at 50 °C, and during heavy shaking and vortexing, for 2 h until all wax had dissolved. The sample was then diluted 100 times, 90 μL transferred to a GC–MS vial, and 10 μL of an internal standard solution (100 ng/μL of tetradecyl acetate, >99% chemical purity, Sigma‐Aldrich, Burlington, MA, USA) added to enable quantification of *E*11,13‐14:Ald *via* comparison of GC peak areas. Samples were analyzed using an Agilent 5977B mass‐selective detector coupled to an Agilent 8890 gas chromatograph equipped with an HP‐INNOWax column (30 m × 0.25 mm ID, 0.25 μm film thickness, J&W Scientific, Folsom, CA, USA). Injector and transfer line temperatures were 260 and 280 °C, respectively, and helium was used as carrier gas at a velocity of 37 cm/s. The oven temperature was set at 58 °C for 1 min after injection and then increased to 230 °C at a rate of 10 °C/min and held for 15 min.

### 2.4 Monitoring survey 2019

Trapping experiments were conducted in strawberry fields in Denmark (*n* = 4) and in Scania (*n* = 3), the southernmost province of Sweden (Supporting Information, Table S1), in 2019 to estimate presence and abundance of *A. comariana* in selected fields. In addition, catch data provided detailed information about the flight periods of the two generations of the pest and aided in planning the timing of the mating disruption experiments the following years. In these surveys and in the subsequent experiments (see below) a matted row system was used with an average row width of 75 cm and an average inter‐row distance of 1.5 m. In the center of each field, two traps with sticky insert were placed at 40 m distance from each other. Traps were checked every week or every second week and inserts replaced if needed (>20 males trapped). Baits were replaced every third week. Experiments were conducted from mid‐May to mid‐November.

### 2.5 Small‐scale mating disruption experiments

Due to lower catches of *A. comariana* in Denmark *versus* Sweden in 2019 (see results), only Swedish crop fields were considered for the mating disruption experiments. The ability of male moths to locate females under mating disruption conditions was measured indirectly by comparing captures of males in pheromone‐baited traps in treatment and control plots within fields, and also *via* survey of larvae in the next generation in such plots. In 2020, five strawberry fields were selected to monitor the first flight period of *A. comariana*. In early June, four traps were placed in the corners of a 20 × 20 square in the center of each field. Traps were checked every week and inserts replaced when needed (>30 males trapped). Trap baits were replaced every 3 weeks. Based on catch data, three fields with high abundance of *A. comariana* were selected for the mating disruption experiment on the second generation of the pest (size and geographic coordinates for these fields are shown in Supporting Information, Table S2). In early September, prior to the application of high‐release dispensers, two 1 ha plots (100 × 100 m) were established in each field, 25 m from the field edge and at least 250 m apart. In the center of each plot, four traps were placed as described above, and each treatment plot had a 10 × 10 matrix of dispensers (a rubber septum attached at 30 cm height to a wooden stick with a needle). Each septum was loaded with 70 mg of *E*11,13‐14:Ald dissolved in heptane, giving a total dose of 7 g per ha.

Mating disruption experiments started the first week of September, a second set of MD dispensers was applied 3 weeks later, and the experiments ended the third week of November. In 2021, three fields in the same area as in 2020 (distance from previous years field: 385–1170 m) were used for a second mating disruption experiment (see Supporting Information, Table S2), with the same general design, that is, plot size and dispenser density, but using a 10 times lower dose of *E*11,13‐14:Ald in dispensers, that is, total dose of 0.7 g per ha. One of the MD plots had to be restricted to 100 × 80 m due to limited size of that field, but had the same number of dispensers. The experiment started the third week of June, a second set of MD dispensers was applied 3 weeks later, and the experiments ended the first week of August. For each experiment, the number of males captured per trap was pooled to give the total number trapped per plot to be used in the statistical analysis. To determine the reduction in trap catches of males in treated plots *versus* control plots (disruption effect), we used the following formula:

$$\%\;\text{disruption}=\left(1-\frac{\text{catches in treatment plot}}{\text{catches in control plot}}\right)x\;100$$

### 2.6 Large‐scale mating disruption experiments

In these experiments, whole strawberry fields were treated with pheromone or left untreated. Four fields in Scania and four fields in Halland were included in the study (Supporting Information, Table S2). The *A. comariana* populations in these fields were monitored during June–July 2023 and 2024 using two traps placed 20 m apart in the center of each field. Based on trap catch data two fields in each region with high catches were selected as treatment and the remaining two fields with lower catches as control. SPLAT droplets (1 g) loaded with 1.5% active ingredient, that is, 15 mg of pheromone, as recommended by the manufacturer, were applied from the plastic tubes directly onto the lower part of the strawberry plants using a caulking gun. In 2023, the density of droplets was 30 per ha and a second application with the same density and dose was done after 3–4 weeks, giving a total dose of 0.90 g per ha of E11–13,14:Ald. In 2024, the treated fields were larger compared to 2023, and the available amount of pheromone smaller, only allowing a single application of SPLAT droplets with similar density as the previous year, that is, a total dose of 0.45 g per ha of *E*11,13–14. In both years, the experiments started the first week of September and ended the first week of November (2023) or the second week of October (2024). Catches were checked every week and inserts replaced if needed. Baits were replaced every fourth week. The disruption effect was estimated in the same way as for the small‐scale experiments, and pooled catches per field were used for the statistical analysis.

### 2.7 Larval surveys

In late May 2021, offspring larvae hatched from overwintering eggs of the autumn generation 2020 were counted in four central rows per plot, with each row divided into three segments and two randomly selected 1 m cells per segment, giving a total of 24 m row analyzed per plot. In late July/early August 2021, offspring larvae produced from the spring generation 2021 were counted with denser sampling compared to the first survey because of lower predicted densities of larvae due to post‐season pruning of plants 1 week earlier. In each plot larvae were counted in 12 rows, with each row divided into three segments, and one randomly selected 1 m cell per segment analyzed, giving a total of 36 m row analyzed per plot. In late May 2024, offspring larvae hatched from overwintering eggs of the autumn generation 2023 were sampled from five rows per field, separated by 15 m. Each row was divided into three segments á 3 m with 15 m distance between segments, giving a total of 45 m row analyzed per field. Data for each field were pooled before analysis.

### 2.8 Attraction of males to 1 g SPLAT droplet *versus* septum lure

An experiment was conducted to check the attraction of males to traps loaded with 1 g SPLAT droplet *versus* traps loaded with the standard rubber septum bait. The amount of *E*11,13‐14:Ald was 150 times higher in a fresh wax droplet (15 mg) compared to a fresh septum (100 μg). The experiment was conducted in a separate strawberry field (55°59.78′ N, 14°16.53′ E) during 4 weeks in September–October 2023. This field was isolated by at least 1700 m from the other fields with ongoing mating disruption. Traps were placed 20 m apart in a row. Five replicates of each lure type were used with every second trap being a SPLAT trap. Captures of moths were checked weekly and inserts replaced if needed. Catches for each week of the experiment were analyzed separately.

### 2.9 Aggregation of males near 1 g SPLAT droplets

In mid September 2024, we observed the flight behavior of male *A. comariana* in one of the SPLAT‐treated fields (Balsby, Supporting Information, Table S2) during an evening with little wind and 20 °C to better understand their attraction to the wax droplets 2.5 weeks after application. In the central part of the field we observed the distribution of males near six droplets in a row and near six droplets in the neighboring row. These 12 droplets were transferred to the top of the vegetation at the position of application prior to the observations. The experiment started at 5:50 pm and ended 75 min later. The observer walked along the transects in total five times, and at each visit at a droplet the number of males on top of the vegetation near (≤0.35 m) a dispenser, or at distances of 1.5, 2.5 or 10 m from the point source, was scored within a 70 × 70 cm square. Each observation period lasted for ≈10 s, and the average number of males at a given droplet‐distance point was used in the statistical analysis.

### 2.10 Attraction of males to conspecific females *versus* septum lure

To better understand how well trap lures can compete with female moths in attracting males, both at disruptive doses of sex pheromone and in a natural environment, we performed an experiment in mid September 2024 to compare catches of males in traps loaded with the standard rubber septum bait or adult female *A. comariana*. Last instar larvae were collected and fed on strawberry tissue in a climate chamber at 22 °C, 70% r.h. and 16:8 L:D regime until pupation, and female pupae were stored under natural temperature and light conditions until adult emergence. Two females 2–6 days old were placed in a glass cylinder (10 × 2.5 cm OD), with the ends covered by cloth, which was placed in the middle of a sticky insert in a trap. A pilot study was performed in an untreated crop field of 18 ha (56°2.63′ N, 14°15.09′ E) earlier in the season (June 20–22), where five female‐baited traps were placed in a central row, two standard monitoring traps with fresh rubber septa as baits were placed in another row 150 m away, and two traps without any lure were placed in a row at the same distance from the female‐baited traps. Traps within each row were separated by 20 m. In the subsequent comparative study, five replicates of each trap type (females *vs* septum) were placed in a pheromone‐treated field (Balsby) and in an untreated field (55°59.78′ N, 14°16.53′ E). Traps were placed along a central row and with 20 m distance between traps, and with every second trap baited with septum. After two nights the traps were removed and catches recorded.

### 2.11 Spermatophore analysis

To evaluate the efficacy of the pheromone treatment in reducing the encounter rate between calling females and mate searching males, the mating frequency of females in SPLAT‐treated fields *versus* untreated fields was compared in 2024. Sampling was performed by walking through the central part of each field and hitting the vegetation with an insect net and catching females flying away. The sampled moths were stored in tubes with ethanol and were later dissected under the microscope to determine the presence of a spermatophore, indicating that the female was mated. Sampling was performed in two treated fields and two untreated fields and data were pooled for each field type prior to analysis.

### 2.12 Statistical analyses

For the mating disruption experiments we used generalized linear models to compare pooled catches between pheromone‐treated plots/fields and their corresponding control plots/fields. Because the catch data were overdispersed, we used a negative binomial distribution with log link function as model. Data from the larval surveys were analyzed with the same model but were assumed to be normally distributed. In the experiments analyzing attraction of males to SPLAT‐baited *versus* septum‐baited traps, we used t‐tests to compare weekly catches in traps with different lures. Catches were log (*x* + 1) transformed prior to the analyses. In the aggregation experiment, the number of males observed at different distances from a SPLAT droplet per route was analyzed using a Kruskal–Wallis test. When comparing attraction of males to female‐baited *versus* septum‐baited traps a Mann–Whitney *U*‐test was applied. Finally, a chi‐square test was used to compare the mating frequency of females in treated fields *versus* untreated fields. All analyses were performed using IBM SPSS statistics v27, New York, NY, USA.

## 3 RESULTS

### 3.1 Flight phenology and moth abundance

Catch data for 2019 indicated two distinct flight periods of *A. comariana* occurring from mid‐June to early August and from early September to early November (Supporting Information, Fig. S1). The flight phenology data were consistent across sites and years. In the area in Scania with fields included in all years of the study, the flight peaked after 190 ± 5 Julian days for the summer generation and after 271 ± 8 Julian days for the autumn generation. As mentioned above, catches in 2019 were lower in Danish fields *versus* Swedish fields (Table S1), and the subsequent mating disruption experiments were thus only performed in Swedish crop fields.

### 3.2 Rubber septa release kinetic study

**Figure 1:**
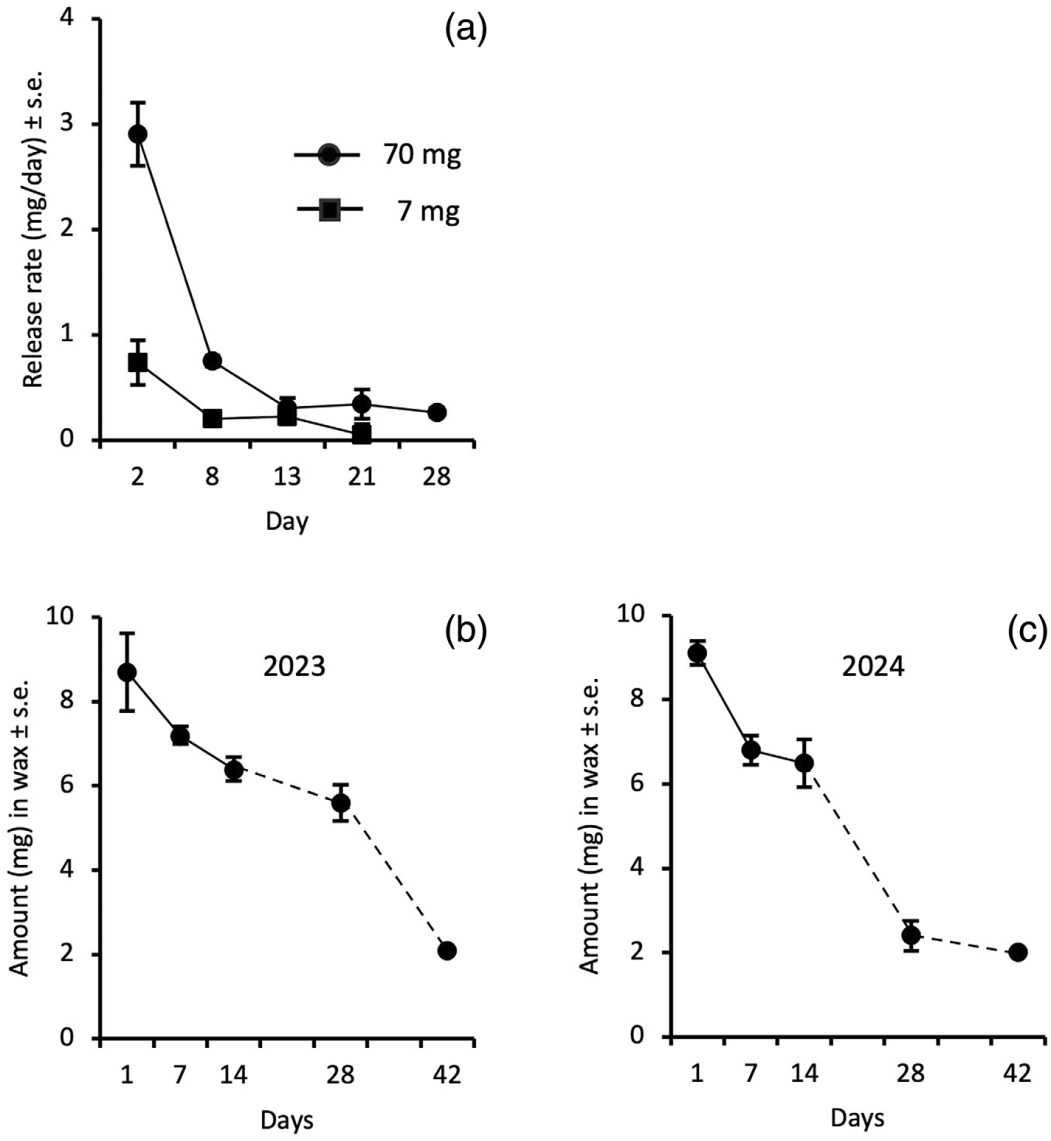
Release data of (*E*)‐11,13‐tetradecadienal, the sex pheromone of *Acleris comariana*, using rubber septa (A) and 1 g SPLAT droplets from 2023 (B) and 2024 (C). Dispensers were aged in a wind tunnel (septa) or under field conditions (wax droplets). In each analysis 2 replicates of dispensers were analyzed.

When rubber septa were loaded with 70 mg of *E*11,13‐14:Ald, the release rate dropped fourfold, from 2.90 to 0.75 mg/day, during the first week, followed by a less pronounced decrease during the next 2 weeks (Fig. 1a). Based on these data, additional dispensers were placed in test fields 3 weeks after the first application. When septa were loaded with 7 mg of *E*11,13‐14:Ald, a fairly stable release rate was obtained for at least 2 weeks (Fig. 1a). The experiment terminated after 21 days when no weight loss of dispensers could be observed.

### 3.3 SPLAT release kinetic study

The estimated concentration of *E*11,13‐14:Ald in one‐day old SPLAT droplets (1.34% in 2023 and 1.27% in 2024) was slightly lower than the predicted value of 1.5% and may be explained by minor loss of the compound during the processing of small amounts of SPLAT by the manufacturer. The release rate of *E*11,13‐14:Ald during the first 2 weeks was similar between years (0.20 mg/day in 2023; 0.18 mg/day in 2024; Fig. 1b,c), but differed greatly during the subsequent 2 weeks (0.29 mg/day in 2023; 0.06 mg/day in 2024), whereas the reversed pattern was observed for the last 2 weeks of the analysis (0.03 mg/day in 2023; 0.25 mg/day in 2024), resulting in similar average release rates for the 6 weeks period. This variation in release rates may be related to temperature differences during the two periods of analysis. After 6 weeks, about 20% of pheromone remained in the droplets.

### 3.4 Small‐scale mating disruption experiments

**Figure 2:**
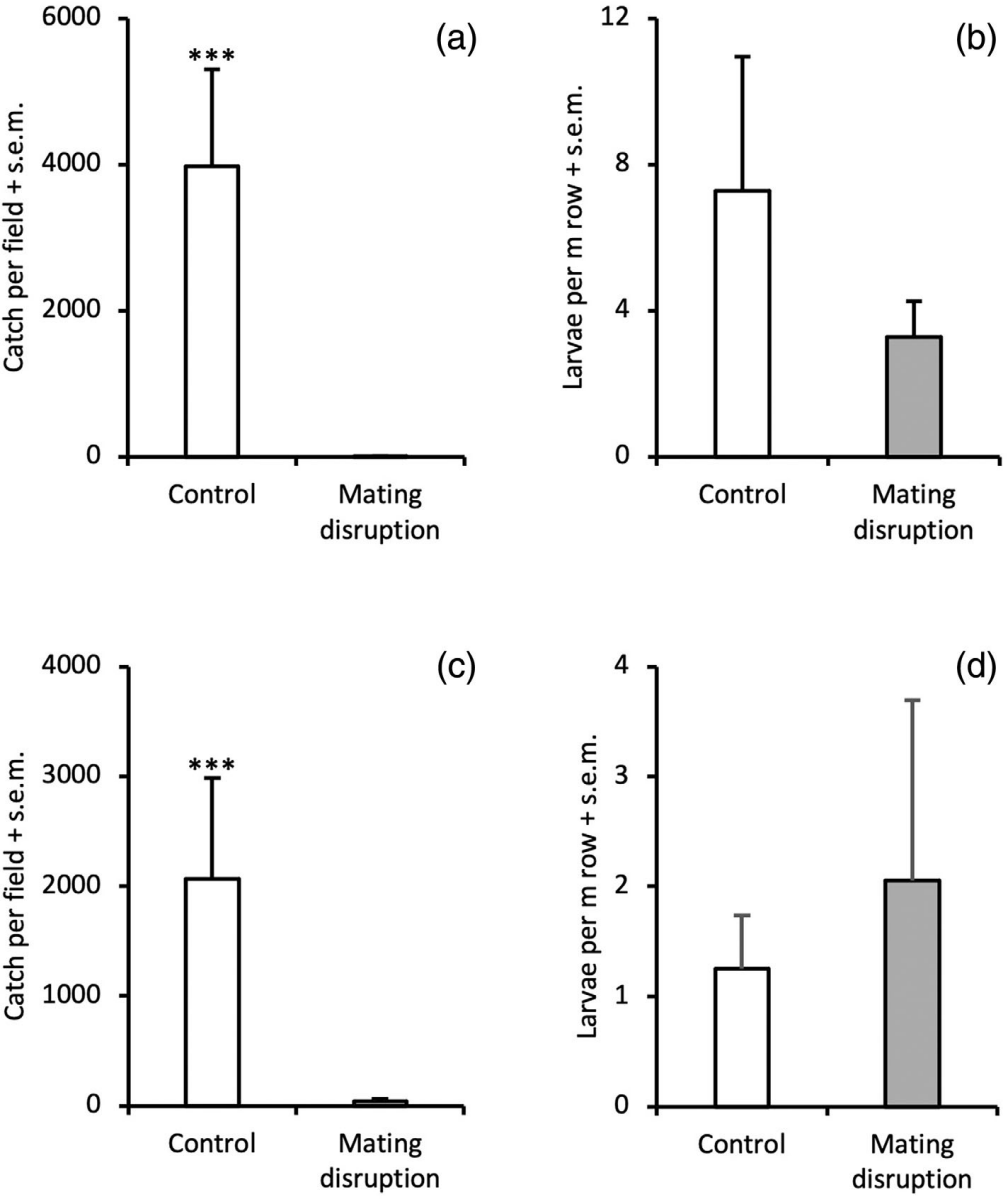
Trap catch of males (A, C) and larval numbers in the subsequent generation (B, D) of *Acleris comariana* in control plots and pheromone‐treated plots in small‐scale mating disruption experiments performed in 2020 and 2021, respectively (*** *P* < 0.001).

In 2020, large variation in abundance of *A. comariana* during the first flight period was observed among the three fields selected for the mating disruption experiment, with highest catches at Nymö (*n* = 3523), followed by Legeved (*n* = 1720) and Viby (*n* = 418). The pattern was similar for catches in the control plots during the second flight period, with lowest catches at Viby (*n* = 1325), but similar catches at Legeved (*n* = 5144) and Nymö (*n* = 5448). In contrast, only seven males were trapped in total in the treatment plots when using rubber septum dispensers at a total dose of 14 g of *E*11,13‐14:Ald per ha, corresponding to >99.9% mating disruption (*χ* ^2^=68.37, d.f. = 1, *P* < 0.001, Fig. 2a). This dramatic decrease in trap catches was, however, not correlated with a subsequent reduction in larval numbers in treated *versus* untreated plots (*χ* ^2^=1.66, d.f. = 1, *P* > 0.05, Fig. 2b).

In 2021, a 10 times lower dose of *E*11,13‐14:Ald was used in septum dispensers in treatment plots, and the experiments were performed during the first flight period of *A. comariana* in fields close to those used in 2020. Catches in control plots in Viby, Legeved and Nymö were 2547, 3358 and 282, respectively, and the corresponding catches in treated plots were 40, 82 and 1, corresponding to 98% disruption effect (*χ* ^2^=22.74, d.f. = 1, *P* < 0.001, Fig. 2c). Similar to the previous year, no difference in larval numbers was observed in treated *versus* untreated plots (*χ* ^2^=0.33, d.f. = 1, *P* > 0.05, Fig. 2d).

### 3.5 Large‐scale mating disruption experiments

**Figure 3:**
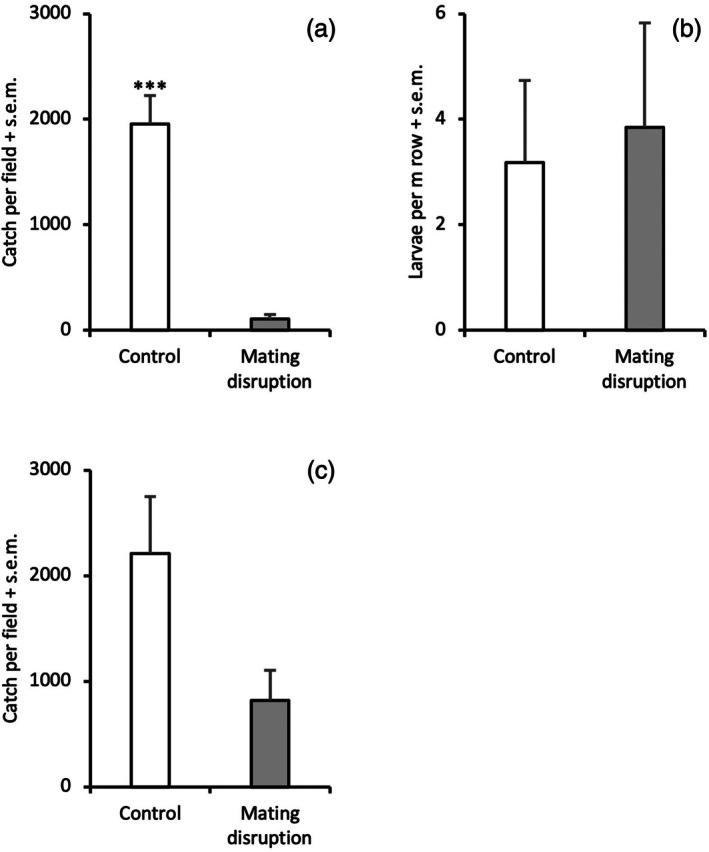
Trap catch of males (A, C) and larval numbers in the subsequent generation (B) of *Acleris comariana* in control fields and pheromone‐treated fields in whole‐field mating disruption experiments performed in 2023 and 2024, respectively (*** *P* < 0.001).

In 2023, whole crop fields were treated with pheromone using SPLAT droplets as dispensers during the autumn. Mean trap catches for the spring generation was 4426 ± 379 in fields selected as control, and 5024 ± 207 in fields selected as treatment. Trap catches during the pheromone treatment were reduced by 95% in treated *versus* untreated fields (*χ* ^2^=17.00, d.f. = 1, *P* < 0.001, Fig. 3a). Again, however, no effect of the treatment was observed in the next generation when analyzing larval numbers in treated *versus* untreated fields (*χ* ^2^=0.71, d.f. = 1, *P* = 0.790, Fig. 3b). In 2024, less pheromone was applied during the autumn compared to the previous year. Mean trap catches for the spring generation was 1134 ± 376 in fields selected as control, and 2436 ± 547 in fields selected as treatment. The disruption effect was much weaker this year, and mean catches were reduced by only 63% in treated fields *versus* untreated fields (*χ* ^2^=1.96, d.f. = 1, *P* = 0.161, Fig. 3c).

### 3.6 Attraction and aggregation of males near 1 g SPLAT droplets

**Figure 4:**
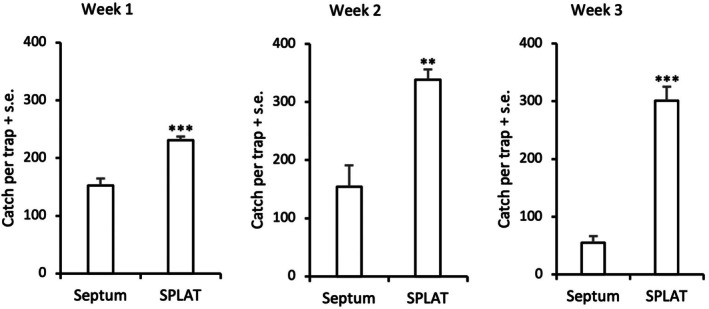
Catch of male *Acleris comariana* in traps baited with rubber septum or SPLAT droplet at different time periods in an untreated crop field (*** *P* < 0.001; ** *P* < 0.01).

**Figure 5:**
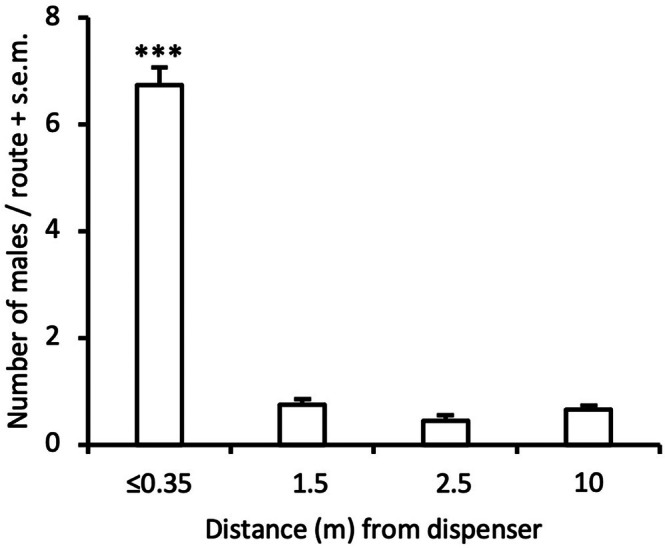
Number of male *Acleris comariana* observed at different distances from SPLAT droplets in a crop field with ongoing mating disruption (*** *P* < 0.001).

In 2023, we observed high catches of *A. comariana* in SPLAT‐baited traps, showing that males could be attracted to such dispensers close enough (<10 cm) to be captured in a trap. Catches in dispenser‐baited traps were significantly higher compared with catches in standard monitoring traps during the first week of the experiment (*t* = 5.03, d.f. = 8, *P* < 0.001) and the effect lasted also for the second (*t* = 3.51, d.f. = 8, *P* < 0.01) and third week (*t* = 7.48, d.f. = 8, *P* < 0.001) of the experiment (Fig. 4). During the last week of the experiment catches were overall much lower, probably due to several nights with frost, and no difference in catches between treatments was observed (data not shown). Although septum‐baited traps attracted fewer *A. comariana* males compared to SPLAT‐baited traps, catches were still high in such traps during the first 3 weeks (22, 22 and eight males/night, respectively) showing that males could still locate septum traps despite nearby high‐dosage dispensers. Our transect observations in 2024 revealed a strong effect of aggregation of males near SPLAT droplets. Males could orient from downwind and land close (≈5 cm) to a droplet, but were never observed on the dispensers. Near (≤0.35 m) such droplets the abundance of males was about 10 times higher compared to just a few meters away from such droplets (*P* < 0.001, Fig. 5).

### 3.7 Attraction of males to conspecific females *versus* septum lure

**Figure 6:**
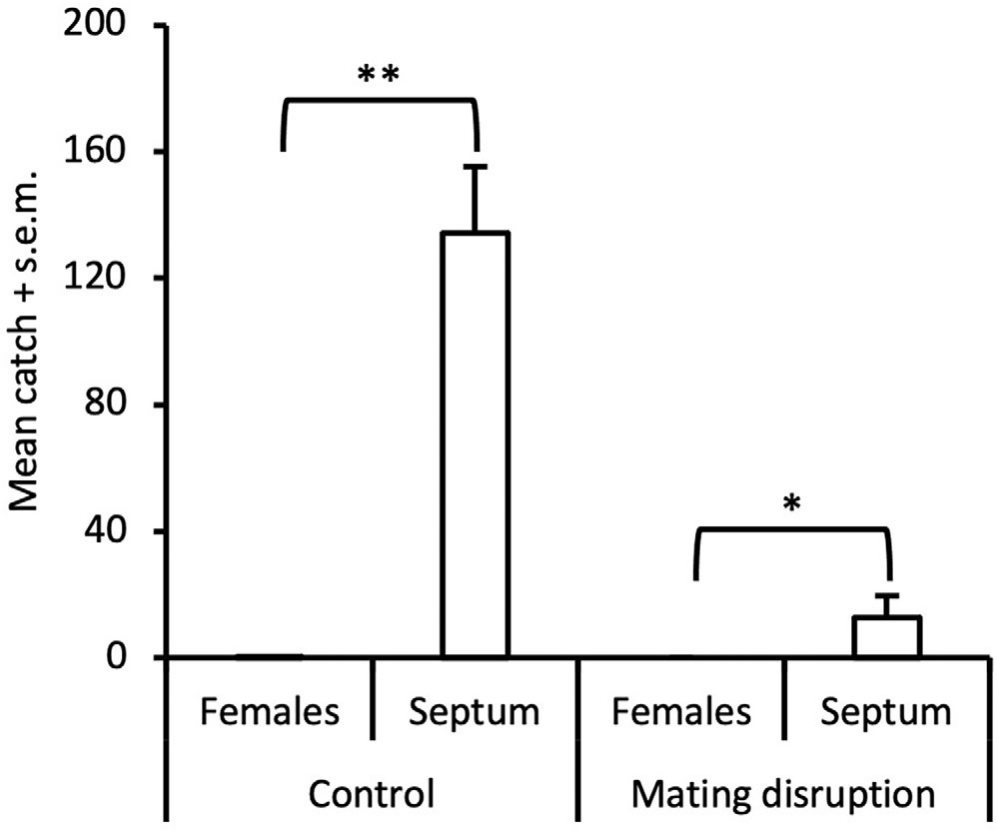
Catch of male *Acleris comariana* in traps baited with two conspecific females or rubber septum and placed in an untreated field (** *P* < 0.01; * *P* < 0.05).

In the pilot study, when female‐baited traps were isolated from septum‐baited traps, catches for two nights were similar between these treatments (female: 127 ± 14 males; septum: 157 ± 8 males), and non‐baited traps did not attract any males, indicating that females can be used as reliable lures in traps. The subsequent experiment, when female‐baited and septum‐baited traps were mixed along the same row, showed that females are outcompeted by the monitoring traps when placed at 20 m distance to such pheromone point sources, both with and without high doses of pheromone, indicting a reduced probability of males in locating females when additional strong point sources are present (Fig. 6). Still, our analysis of field‐collected moths revealed no difference in mating frequency of females in SPLAT‐treated (75%, *n* = 20) *versus* untreated fields (74%, *n* = 19) (*χ* ^2^=0.01; d.f. = 1; *P* > 0.05).

## 4 DISCUSSION

This study shows the first case where pheromone‐based mating disruption has been targeting a species within the moth genus *Acleris*. Our results suggest that the method may be an efficient strategy to control the strawberry pest *A. comariana* but that further optimization is needed. Despite high densities of the species in the study fields, as revealed by high trap catches in the previous generation, attraction of males to traps was reduced by ≥95% in the experiments in 2020, 2021 and 2023. A corresponding reduction in larval numbers in the subsequent generation was not achieved, however, showing that a drastic reduction in trap catch in treated areas can be a weak predictor of successful mating disruption. Several factors may have contributed to the lack of a disruption effect. Because *E*11,13‐14:Ald is expensive to purchase and synthesize, the amounts available for our experiments were limited, even with our own batches synthesized. The large sizes of suitable strawberry fields in combination with restricted amounts of pheromone resulted in lower doses applied per ha (0.45–14 g) compared to similar studies on other tortricid pests.^20^, ^21^, ^22^, ^23^, ^24^, ^25^ For example, SPLAT droplets used for control of *Epiphyas postvittana* (Walker)^24^ and *G. molesta* ^26^ used 5–96 and 160 g/ha of pheromone, respectively. The fact that *A. comariana* occurred at high densities in many study fields further limited the effect of the treatment as competitive disruption was the main mechanism causing reduced catches in traps. The release of pheromone from SPLAT droplets in this study was similar to that observed for *E. postvittana* ^24^ with <30% of pheromone remaining in the wax after 6 weeks.

Most studies on mating disruption in moth pests follow a similar protocol to evaluate the effect of the treatment, which includes catches of males in assessment traps, assumed to simulate calling females, and one or several parameters to estimate damage on the crop.^27^ Experiments analyzing the actual mechanism(s) that trigger disruption are rarely performed, which limits further optimization of this control method for a given pest‐crop system. There is large variation in sensitivity to the pheromone treatment between moth species, even at family level, which may affect which mechanism causes disruption of a pest at a given dose of disruptant. For example, experiments on *G. molesta* showed that this pest was disrupted competitively when using low‐release dispensers, but shifted to non‐competitively when high‐release dispensers were applied.^28^ Such shift was not observed for *C. pomonella* when high‐release dispensers (aerosol emitters) were used, indicting that this species is disrupted competitively regardless of release rate of pheromone from dispensers.^29^ In this study, we performed experiments on *A. comariana* to better understand the lack of correlation between trap catch of males during pheromone treatment and abundance of larvae in the next generation. When comparing catches of males in traps baited with conspecific females or rubber septa, males clearly preferred septum‐baited traps, both with and without high doses of pheromone. In addition, males preferred SPLAT‐baited traps *versus* septum‐baited ones. Because males were attracted and got captured in SPLAT‐baited traps, their olfactory sense was not impaired and the drastic reduction of trap catches in treated areas could be explained by competitive disruption. In addition, males aggregated near the dispensers and few males were observed just a few meters away from these, strongly indicating that induced allopatry was the main mechanism causing the disruption effect.

We observed strong attraction of male *A. comariana* to SPLAT droplets. The recommended dose of 1.5% active ingredient in the wax was used in these experiments, and the amount of pheromone was initially 150 times higher in such dispenser compared to a septum bait. Surprisingly, significantly higher catches were observed in SPLAT‐baited *versus* septum‐baited traps during the first week, and the effect became stronger during the second and third week (Fig. 4). In a previous dose–response experiment we showed that males were highly attracted to septa loaded with 1 mg of *E*11,13‐14:Ald, demonstrating that they can tolerate high doses of the pheromone although females do not seem to produce large amounts of the active compound.^15^ In the study on *G. molesta*, traps baited with a SPLAT droplet attracted very few males during the first 17 days of the mating disruption experiment, but later became equally attractive to traps baited with a septum lure.^26^ In that study, the release rate of the pheromone from dispensers was 0.51 mg/h during the first 2 weeks, but during the subsequent 50 days it dropped to 0.23 mg/h, matching the release rates observed during the first weeks in the present study, indicating that males of *A. comariana* and *G. molesta* show similar sensitivity to their sex pheromones. Attraction of *G. molesta* as well as *Choristoneura rosaceana* (Harris) and *Argyrotaenia velutinana* (Walker) has been observed also to high‐dosage polyethylene tube dispensers.^30^

The efficacy of the pheromone treatment under conditions of competitive disruption will depend on the relative abundance, distribution, and strength of the three types of point sources available for males, that is, conspecific females, traps and dispensers. In the current study, the low catch of males in the monitoring traps in treated areas was probably because these traps were outcompeted by the more abundant and more attractive disruption dispensers. In addition, the density of calling females was much higher than the density of dispensers, making it possible for males to locate females despite their stronger attraction to dispensers. The combination of these factors resulted in high frequencies of mated females in spite of low trap catches in treated fields. In the experiments performed in 2024, only a single application of dispensers was performed, resulting in a lower total dose of pheromone used compared to the year before, which resulted in higher trap catches in treated fields. Thus, to achieve efficient control of *A. comariana* a denser matrix of high‐release dispensers is needed to increase the effect of competitive disruption. In addition, ways to reduce the initial population size of the pest in the crop fields are also important, since competitive disruption is difficult to achieve when pest densities are high.

So far, the sex pheromone has been identified in five pest species of genus *Acleris*: *A. comariana*, *Acleris fimbriana*,^31^ *Acleris gloverana*,^32^ *Acleris minuta* ^33^ and *Acleris variana*.^34^ All species use *E*11,13‐14:Ald as pheromone component, indicating a potential for this compound to be used broadly for mating disruption of *Acleris* pests. When considering *A. comariana* the dose of *E*11,13‐14:Ald applied in crop fields has to increase to achieve efficient mating disruption, but the high cost of producing the compound may be a limiting factor for cost‐effective control of the pest. One way to lower the cost would be to produce a mixture of E and Z isomers of the compound, which are often significantly cheaper than producing pure isomers. Using such mixture would not compromise the efficiency of the control method, as *A. comariana* males are equally attractive to various ratios of the two isomers and also to pure *Z*11,13‐14:Ald.^15^

The lack of detected disruption effect in the small‐scale experiments could be a consequence of treating a too small fraction (<10%) of the crop field and allowing females to mate in the remaining part of the field and disperse into the treated area for egg laying. We observed individuals flying approximately 50 m within less than a minute during wind still, warm and sunny conditions (GP Svensson and V Tönnberg, unpublished). This observed characteristic implies difficulties with achieving population control due to potential inflights from surrounding areas. A weak correlation between trap catch reduction and subsequent damage has been observed in several studies with similar design of the mating disruption experiment.^35^, ^36^, ^37^ When treating whole fields, such effect is reduced, but dispersal could still occur from nearby untreated fields. In our study area the distance between strawberry fields was sometimes less than a few hundreds of meters, and with a high dispersal potential for the pest, all fields within an area have to be pheromone‐treated so the effect of immigrating mated females becomes negligible. Our own dispersal study on *A. comariana* using pheromone traps detected captures up to 1.6 km from a focal field (GP Svensson and V Tönnberg, unpublished), indicating good dispersal capacity, and/or that alternative hosts occur in the landscape, which can maintain a small population of the species. Identifying and removing additional host species for *A. comariana* near crop fields, for example *Potentilla palustris* and raspberry,^8^, ^38^ and facilitating conditions for its natural enemies,^5^ may be important when developing integrated pest management tactics for this strawberry pest.

## 5 CONCLUSION

Our results on *A. comariana* add to previous studies suggesting competitive disruption as the main mechanism causing reduced trap catches in pheromone‐based control trials in moth pests.^17^ Understanding how the density and distribution of calling females *versus* high‐release dispensers will affect the ability of male moths to locate conspecific females for mating is still not fully understood but is key for the development of robust systems for mating disruption in agricultural crop systems. The combination of an expensive sex pheromone, large size of the crop fields, and large populations sizes in many fields makes *A. comariana* particularly challenging in this context. With further optimization of the method, however, successful mating disruption of this important strawberry pest may be possible.

## CONFLICT OF INTEREST

The authors declare that they have no conflict of interest.

## Supporting information


**Figure S1.** Trap catch data of male *Acleris comariana* from the Viby field in 2019 indicating two distinct flight periods of the pest. The total catch over the season is indicated. The same flight pattern was shown in all years and sites included in this study.

**Table S1.** Field sites for trapping experiments of *Acleris comariana* in 2019.

**Table S2.** Crop fields included in this study, which were treated with pheromone for mating disruption (MD) or left untreated (control). In 2020–2021, a 1 ha square of a field was pheromone‐treated, and the rest of the field was used as control (MD/control). In 2023–2024, whole fields where either pheromone‐treated of left untreated.

**Data S1.** Supporting Information.

## Data Availability

The data that support the findings of this study are available from the corresponding author upon reasonable request.
