# Supplementary figures and images for: Induced allopatry as main mechanism explaining trap catch reduction in low dose mating disruption trials on the strawberry pest Acleris comariana (Lepidoptera: Tortricidae)
Source: Pest Manag Sci. 2025 May 9;81(9):5224–33. doi: 10.1002/ps.8877 (PMC12332102; doi:10.1002/ps.8877)

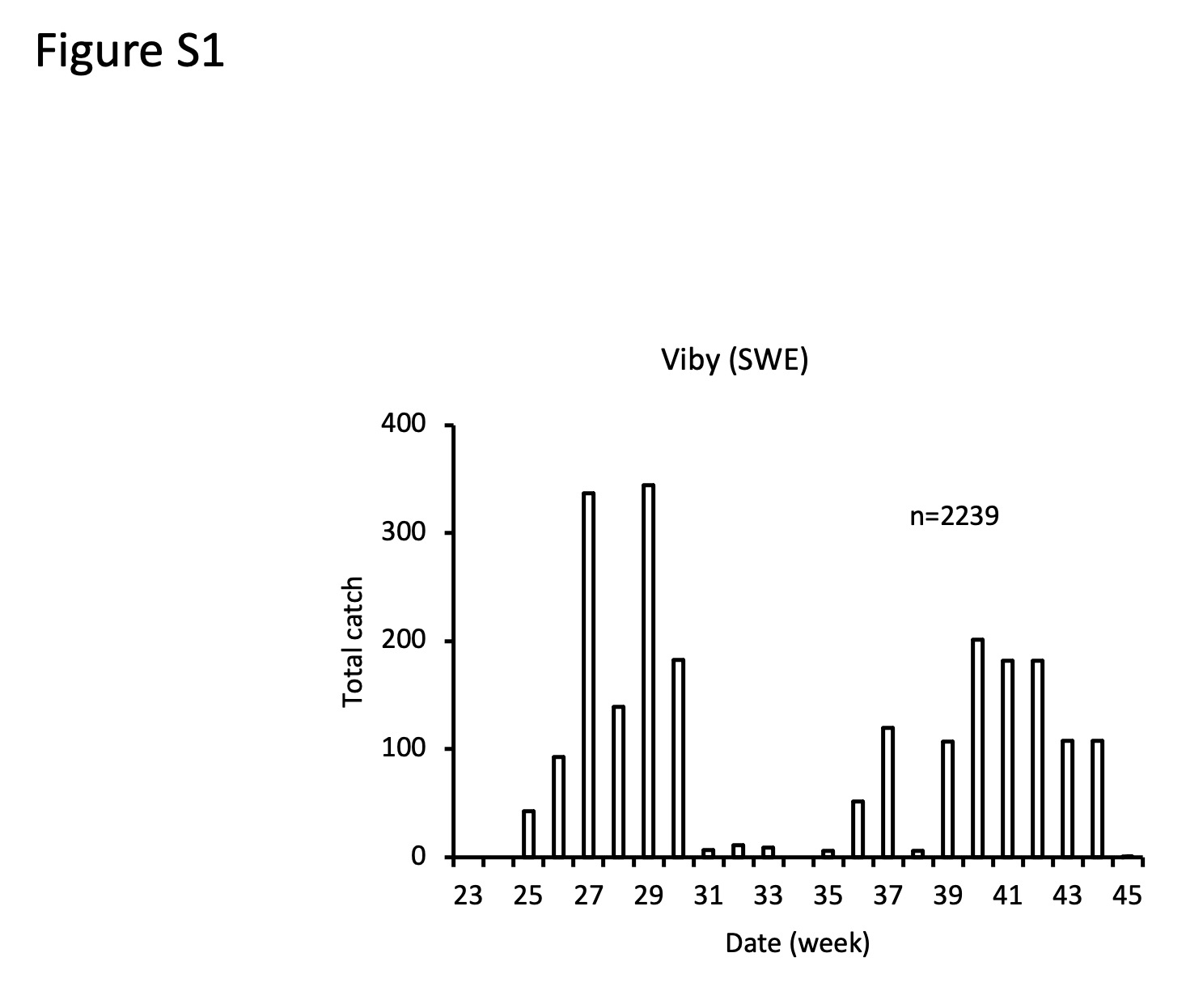

Supplement: Supplementary file 1 — Figure S1. Trap catch data of male Acleris comariana from the Viby field in 2019 indicating two distinct flight periods of the pest. The total catch over the season is indicated. The same flight pattern was shown in all years and sites included in this study. [file PS-81-5224-s002.jpg]
