# Supplementary material for: Induced allopatry as main mechanism explaining trap catch reduction in low dose mating disruption trials on the strawberry pest Acleris comariana (Lepidoptera: Tortricidae)
Source: Pest Manag Sci. 2025 May 9;81(9):5224–33. doi: 10.1002/ps.8877 (PMC12332102; doi:10.1002/ps.8877)
Supplement: Supplementary file 2 — Table S1. Field sites for trapping experiments of Acleris comariana in 2019. [file PS-81-5224-s003.docx]

**Table S1:** Field sites for trapping experiments of *Acleris comariana* in 2019.

Site Country Geographic coordinates Total catch

Borgeby Sweden 55°44.15'N 13°3.54'E 1111

Esrum Denmark 56°1.80'N 12°17.02'E 422

Jordbærgården Denmark 55°43.03'N 12°17.70'E 448

Kildebrønde Denmark 55°36.24'N 12°16.06'E 447

Nymö Sweden 56°0.94'N 14°18.93'E 2000

Ventegodt Denmark 55°31.20'N 12°8.21'E 33

Viby Sweden 56°1.07'N 14°15.14'E 2239
