# Supplementary material for: Induced allopatry as main mechanism explaining trap catch reduction in low dose mating disruption trials on the strawberry pest Acleris comariana (Lepidoptera: Tortricidae)
Source: Pest Manag Sci. 2025 May 9;81(9):5224–33. doi: 10.1002/ps.8877 (PMC12332102; doi:10.1002/ps.8877)
Supplement: Supplementary file 3 — Table S2. Crop fields included in this study, which were treated with pheromone for mating disruption (MD) or left untreated (control). In 2020–2021, a 1 ha square of a field was pheromone‐treated, and the rest of the field was used as control (MD/control). In 2023–2024, whole fields where either pheromone‐treated of left untreated. [file PS-81-5224-s004.docx]

**Table S2**. Crop fields included in this study, which were treated with pheromone for mating disruption (MD) or left untreated (control). In 2020-2021, a 1 ha square of a field was pheromone-treated, and the rest of the field was used as control (MD/control). In 2023-2024, whole fields where either pheromone-treated of left untreated.

Year(s) Site County Geographical coordinates Area (ha) Treatment

2020 Nymö 1 Skåne 56°0.94'N 14°18.93'E 10 MD/control

2020 Legeved 1 Skåne 56°1.34'N 14°17.60'E 12 MD/control

2020+2021 Viby Skåne 56°1.07'N 14°15.14'E 11 MD/control

2021 Legeved 2 Skåne 56°1.32'N 14°17.18'E 15 MD/control

2021 Nymö 2 Skåne 56°1.30'N 14°19.84'E 8 MD/control

2023 Garden 1 Halland 57°4.17'N 12°18.03'E 4 Control

2023 Jonstaka Halland 57°4.80'N 12°16.96'E 4 MD

2023+2024 Kanaan Skåne 56°1.43'N 14°15.31'E 10 Control

2023 Lennartsson Halland 57°5.71'N 12°17.82'E 5 MD

2023 Magasinsvången Skåne 56°7.00'N 14°17.76'E 16 Control

2023+2024 Nymö 3 Skåne 56°1.01'N 14°18.80'E 8 Control+MD

2023 Vänster markväg Skåne 56°0.81'N 14°16.79'E 9 MD

2023 Wilhelmina Halland 57°5.19'N 12°17.84'E 6 Control

2024 Balsby Skåne 56°3.96'N 14°12.88'E 18 MD

2024 Garden 2 Halland 57°4.08'N 12°17.96'E 4 MD

2024 Johnssons Halland 57°4.04'N 12°18.57'E 4 MD

2024 Kalle Thim Skåne 56°0.13'N 14°24.34'E 10 MD

2024 Kvarnen Halland 57°5.31'N 12°17.81'E 2 Control

2024 Tostes Halland 57°5.54'N 12°18.26'E 5 Control
