# Supplementary material for: Induced allopatry as main mechanism explaining trap catch reduction in low dose mating disruption trials on the strawberry pest Acleris comariana (Lepidoptera: Tortricidae)
Source: Pest Manag Sci. 2025 May 9;81(9):5224–33. doi: 10.1002/ps.8877 (PMC12332102; doi:10.1002/ps.8877)
Supplement: Supplementary file 4 — Data S1. Supporting Information. [file PS-81-5224-s001.docx]

**Supplementary information**

*Description of the synthesis*

(*E*)-Tetradeca-11,13-dienal (***E*11,13-14:Ald**) was prepared from 1,10-decanediol in 30% total yield (Scheme 1). 1,10-Decanediol was transformed into the THP-protected bromo alcohol **1** in 80% yield, in a two-step sequence, using the procedures of Chong et al.^1^ and Caraballeira et al.^2^ The diol was mono-brominated, using 48% HBr, followed by THP-protection of the bromo alcohol, using DHP under catalytic acidic conditions. *(E)*-Buta-1,3-dienyl diethyl phosphate (**4**) was prepared according to the procedure of Cahiez et al.^3^ from crotonaldehyde (**2**) and diethyl chlorophosphate (**3**) in 76% yield and good isomeric purity, *E*:*Z* = 97.5:2.5, determined by GC. (*E*)-tetradeca-11,13-dien-1-ol (**5**), was prepared from bromide **1**, via formation of a Grignard reagent, which was coupled to dienylphosphate **4**, to give the THP-protected alcohol intermediate, according to the procedure of Cahiez et al.^3^ for similar compounds. The THP-group was removed from the coupling product under acidic conditions according to the procedure from Caraballeira et al.,^2^ for similar compounds, to give the dienol **5** in 60% overall yield. The isomeric purity, *E*:*Z* = 93:7, was determined from ^1^H NMR (see Experimental). No efforts were made for *E*/*Z*-separations on GC. (*E*)-Tetradeca-11,13-dienal (***E*11,13-14:Ald**) was prepared in 81% yield, by oxidation of dienol **5** with PCC/silica/molecular sieves 3 Å, a modified procedure of Gung and Dickson^4^ for similar compounds. The isomeric purity of aldehyde **6** was maintained from alcohol **5**, *E*:*Z* = 93:7, and was determined from ^1^H NMR (see Experimental). No efforts were made for *E*/*Z*-separations on GC.

**References**

1. Chong JM, Heuft and Rabbat P, *J Org Chem* **65**: 5837–5838 (2000)

2. Carballeira NM, Nashbly N and Padilla LF, *Chem Phys Lipids* **145**: 37–44 (2007)

3. Cahiez G, Hablak and Gagaer O, *Org Lett* **10**: 2389–2392 (2008)

4. Gung BW and Dickson H, *Org* *Lett* **4**: 2517–2519 (2002)

**Scheme 1**. (**a**) 1. 48% HBr, toluene, reflux. 2. DHP, p-TSA·H2O, CH_2_Cl_2_ (80% yield). (**b**) 1. Aldehyde **2**, *tert*-BuOK, THF, −78 °C. 2. Add phosphate **3**, −78 °C to 20 °C (76% yield). (**c**) 1. Bromide **1**, Mg, THF, room teperature. 2. Grignard solution of **1** transferred to phosphate **4**, Fe(acac)_3_, THF, room temperature. 3. p-TSA·H_2_O, MeOH, room temperature (60% yield). (**d**) PCC/silica/MS 3 Å, CH2Cl2, room temperature (81% yield).

**Experimental**

Dry CH_2_Cl_2_ and THF was obtained from a solvent purification system (Activated alumina columns, Pure Solv PSMD-5). All other chemicals were used as received. Merck Silica gel 60 (0.040-0.063 mm, 230-400 mesh ASTM) was used in preparative liquid chromatography, using an increasing gradient (1-100%) of distilled EtOAc in distilled cyclohexane. Thin layer chromatography was performed on silica gel plates (Merck 60, pre-coated aluminium foil) to monitor the progress in reactions and was developed in UV-light and/or sprayed with vanillin in sulphuric acid followed by heating with a heat gun. Conversion analyses and purity of products were checked with GC analyses on a Varian 3300 GC instrument equipped with a flame ionization detector (FID) using a capillary column Hp-5 (30 m× 0.25 mm id, df =0.25 μm, with nitrogen (9 psi) as the carrier gas and a split ratio of 1:20). The oven temperature was programmed at 100 °C for 2 min followed by a gradual increase of 10 °C/min to reach a final temperature of 300 °C, with 5 min hold time. Mass spectra were recorded on a Hewlett-Packard 6890N GC, equipped with a capillary column vf-5 ms (30 m× 0.25 mm i.d., df = 0.25 μm), and coupled to a HP 5973 MS-detector in electron impact (EI, 70 eV) ionization mode. The oven temperature was programmed at 100 °C for 2 min followed by a gradual increase of 10 °C/min to reach a final temperature of 300 °C, with 5 min hold time. NMR spectra were recorded on a Bruker Avance 500 (500 MHz ^1^H, 125.8 MHz ^13^C) instrument and all shifts are reported in ppm and CDCl_3_ residual peak was used as reference, 7.26 (^1^H) and 77.16 (^13^C) ppm, respectively.

**10-Bromo-1-[(tetrahydropyran-2-yl)oxy]decane (1)**

A solution of HBr (46.5 ml, 413 mmol, 48 wt% in H_2_O) and 1,10-decanediol (60.0 g, 344 mmol) in toluene (600 ml) was refluxed for 6 days. After cooling to room temperature, the aqueous phase was removed and the organic phase was washed with 2M aqueous NaOH (50 ml), brine (50 ml) and finally dried over MgSO_4_. The toluene solution containing bromo alcohol was applied on a flash column with silica gel (200 g), using EtOAc/cyclohexane as eluent. Flash chromatography was repeated twice with mixed fractions. Finally, the bromo alcohol was isolated as a clear light-yellow oil, 73.1 g (90% yield, 99.1% purity according to GC) and checked by NMR.

3,4-Dihydro-*2H*-pyran (DHP, 36.4 ml, 399 mmol) was added dropwise to a solution of the bromo alcohol (72.8 g, 307 mmol), from above, and p-TSA·H_2_O (380 mg, 2 mmol) in CH_2_Cl_2_ (200 ml). The reaction mixture, a clear solution, was stirred at room temperature for 23 hours and turned black during that time. The reaction mixture was washed with H_2_O (2 x 100 ml), Aqueous saturated NaHCO_3_ (3 x 100 ml), and the organic phase was then dried over MgSO_4_. Evaporation of solvent resulted in a brown oil (106.5 g, 92% purity according to GC), which was purified by repeated flash chromatography (silica gel, EtOAc/cyclohexane). The title compound **1** was isolated as clear light-yellow oil, 79.2 g (80% yield, 96.6% purity according to GC).

^1^H NMR (500 MHz, CDCl_3_): 1.30-1.45 (12H, m), 1.48-1.62 (6H, m), 1.68-1.74 (1H, m), 1.80-1.88 (3H, m), 3.36-3.41 (3H, m), 3.48-3.52 (1H, m), 3.72 (1H, dt, *J* = 9.6, 6.9 Hz), 3.87 (1H, ddd, *J* = 11.0, 7.6, 3.2 Hz), 4.57 (1H, dd, *J* = 4.3, 2.9 Hz) ppm. ^13^C NMR (125.8 MHz, CDCl_3_): 19.88, 25.71, 26.39, 28.33, 28.90, 29.52, 29.58, 29.61, 29.93, 30.99, 33.02, 34.05, 62.49, 67.82, 99.03 ppm. MS (EI) *m/z* (relative intensity): 321 (2), 319 (2), 163 (1), 137 (2), 115 (2), 101 (8), 85 (100), 56 (17), 41 (12). All spectral data are in accordance with reported data in the literature.^1^

***(E)*-Buta-1,3-dienyl diethyl phosphate (4)**

Crotonaldehyde (**2**) (predominantly *trans*, ≥99%, Aldrich, 21.0 ml, 253 mmol) was added dropwise (20 minutes) to a supension of *tert*-BuOK (34.1 g, 304 mmol) in THF (400 ml) at −78 °C, under an argon atmosphere, keeping the reaction temperature below −60 °C during the addition. After 1 hour, diethyl chlorophosphate (**3**) (40.0 ml, 277 ml) was added dropwise (20 minutes), keeping the reaction temperature at −60 °C during the addition. After addition the cooling bath was removed, and the mixture was allowed to reach room temperature. After 2 hours the reaction was quenched with 1 M HCl (500 ml) and the mixture was extracted with EtOAc (4 x 100 ml). The combined organic phases were washed with brine (250 ml) and dried over MgSO_4_. Evaporation of solvent resulted in a yellow/orange turbid oil (65.0 g, 87% purity according to GC), which was purified by repeated flash chromatography (silica gel, EtOAc/cyclohexane). The title compound **4** was isolated as clear yellow oil, 39.8 g (76% yield, 97.2% purity and *E*:*Z* = 97.5:2.5 according to GC).

GC: t_R_(*Z*, minor isomer) 7.18 min and t_R_(*E*, major isomer) 7.52 min. ^1^H NMR (500 MHz, CDCl_3_): 1.36 (6H, t, *J* = 7.6 Hz), 4.13-4.21(4H, m), 5.06 (1H, d, *J* = 10.2 Hz), 5.18 (1H, d, *J* = 16.9 Hz), 6.04 (1H, t, *J* = 11.5 Hz), 6.21 (1H, ddd, *J* = 16.9, 11.0, 10.2 Hz), 5.06 (1H, dd, *J* = 11.9, 6.5 Hz) ppm. ^13^C NMR (125.8 MHz, CDCl_3_): 16.20 (d, *J_P-C_* = 6.7 Hz), 64.66 (d, *J_P-C_* = 5.8 Hz), 117.11, 118.33 (d, *J_P-C_* = 10.5 Hz), 131.18, 139.91 (d, *J_P-C_* = 5.6 Hz) ppm. MS (EI) *m/z* (relative intensity): *E*-isomer (major isomer), 206 (M^+^, 92), 178 (13), 155 (69), 150 (31), 127 (80), 109 (81), 99 (100), 91 (28), 81 (85), 70 (49), 52 (31), 29 (14). *Z*-isomer (minor isomer) showed similar fragmentation as the *E*-isomer. All spectral data are in accordance with reported data in the literature.^2^

**(*E*)-tetradeca-11,13-dien-1-ol (5)**

Bromide **1** (39.5 g, 123 mmol) was added dropwise (50 min) to Mg-turnings (12.4 g, 511 mmol) and a few crystals of iodine in THF (480 ml), at room temperature under an argon atmosphere. After 1.5 hours the bromide **1** was consumed and the formed Grignard reagent was transferred to a flask with dienyldiethtylphosphate **4** (20.0 g, 102 mmol) and Fe(acac)_3_ (366 mg, 1 mmol) in THF (130 ml), at room temperature under an argon atmosphere. After 1 hour, the reaction mixture was quenched with 1 M HCl (250 ml). The organic phase was separated, and the aqueous phase was extracted with Et_2_O (3 x 100 ml). The combined organic phases were washed with brine (100 ml) and dried over MgSO_4_. Evaporation of solvent resulted in a brown turbid oil (THP-protected dienol, 43.5 g)

The crude product from above, combined with a crude product from an identical reaction, 80.5 g in total, and p-TSA·H_2_O (1.91 g, 10 mmol) was dissolved in MeOH (500 ml) and stirred at room temperature for 18 hours. MeOH was removed under reduced pressure and the residue was washed with 10% NaHCO_3_ (200 ml). The organic phase was separated, and the aqueous phase was extracted with Et_2_O (8 x 100 ml). The combined organic phases were washed with brine (200 ml) and dried over MgSO_4_. Evaporation of solvent resulted in a red/brown clear oil, 45.2 g. Impurities in the crude product were removed by distillation (up to 75 °C / 1 mbar), and the residue from the distillation was further purified by flash chromatography (silica gel, EtOAc/cyclohehane). The title compound **5** was obtained as a clear yellow oil, 22.3 g [98.0% purity according to GC and *E*:*Z* = 93:7 according to ^1^H NMR. 60% yield based on the total amount of dienyldiethtylphosphate 4 (36.3 g, 176 mmol) in the reactions].

^1^H NMR (500 MHz, CDCl_3_): 1.26-1.40 (16H, bs), 1.47 (1H, m), 2.07 (*E,* 1.86H, q, *J* = 7.1 Hz), 2.16 (*Z*, 0.14H, m) 3.64 (2H, t, *J* = 6,6 Hz), 4.95 (*E,* 0.93H, d, *J* = 10.1 Hz), 5.08 (*E,* 1H, d, *J* = 16.9 Hz, overlapping with *Z*), 5.17 (*Z*, 0.07H, d, *J* = 15.7 Hz), 5.43-5.48 (0.07H, m),5.70 (*E,* 0.93H, dt, *J* = 15.2, 7.3 Hz), 6.04 (*E,* 1H, dd, *J* = 15.0, 10.6 Hz, overlapping with *Z*), 6.31 (*E,* 0.93H, dt, *J* = 17.0, 10.3 Hz), 6.64 (*Z,* 0.07H, dt, *J* = 17.0, 10.6 Hz) ppm. ^13^C NMR (125.8 MHz, CDCl_3_): 25.90, 29.34, 29.57, 29.61, 29.66, 29.73, 32.69, 32.99, 63.25, 114.67, 131.03, 135.73, 137.31 ppm. MS (EI) *m/z* (relative intensity): 210 (M^+^, 7), 192 (2), 163 (2), 149 (2), 135 (10), 121 (16), 109 (18), 95 (44), 81 (72), 67 (100), 54 (60), 41 (42). All NMR spectral data are in accordance with reported data in the literature for a similar compound, (*E*)-dodeca-11,13-dien-1-ol,^2^ and MS data are in accordance with the literature.^3^

**(*E*)-Tetradeca-11,13-dienal (*E*11,13-14:Ald)**

Dienol **5** (22.3 g, 106 mmol), dissolved in CH_2_Cl_2_ (100 ml), was added dropwise (10 minutes) to a suspension of PCC (34.3 g, 159 mmol), silica gel (35 g) and molecular sieves (3 Å, 10 g) in CH_2_Cl_2_ (350 ml) at room temperature under an argon atmosphere. After 3.5 hours, Et_2_O (250 ml) was added and the mixture was filtrated through a plug of silica gel in a glass filter funnel and rinsed with another Et_2_O (750 ml). The green-coloured filtrate was once more filtrated through a plug of silica and the filtrate was yellow. Evaporation of solvent resulted in a green/brown oil, 21.0 g (94.5% purity according to GC). The crude product was purified by flash chromatography (silica gel, EtOAc/cyclohexane) and the title compound ***E*11,13-14:Ald** was obtained as a slightly yellow clear oil, 17.8 g (98.8% purity according to GC and *E*:*Z* = 93:7 according to ^1^H NMR, in 81% yield).

^1^H NMR (500 MHz, CDCl_3_): 1.26-1.42 (12H, m,), 1.62 (2H, quintet, *J* = 7.1 Hz), 2.07 (*E,* 1.86H, q, *J* = 7.1 Hz), 2.17 (*Z,* 0.14H, app q, *J* ̴ 7.1 Hz), 2.42 (2H, dt, *J* = 1.7, 7.3 Hz), 4.95 (*E,* 0.93H, d, *J* = 10.1 Hz), 5.08 (*E,* 1H, d, *J* = 17.0 Hz, overlapping with *Z*), 5.17 (*Z,* 0.07H, d, *J* = 16.8 Hz), 5,45 (*Z,* 0.07H, m), 5.70 (*E,* 0.93H, dt, *J* = 15.2, 7.7 Hz), 5.99 (*Z,* 0.07H, t, *J* = 11.3 Hz), 6.04 (*E,* 0.93H, dd, *J* = 14.9, 10.7 Hz), 6.31 (*E,* 0.93H, dt, *J* = 17.0, 10.3 Hz), 6.64 (*Z,* 0.07H, dt, *J* = 16.9, 10.6 Hz), 9.76 (*E* ,1H, t, *J* = 1.8 Hz, overlapping with *Z*) ppm. ^13^C NMR (125.8 MHz, CDCl_3_): 22.22, 29.30, 29.48, 29.49, 29.54, 32.68, 44.07, 114.75, 131.01, 135.73, 137.50, 203.13 ppm. MS (EI) *m/z* (relative intensity): 208 (M^+^, 10), 193 (0.4), 179 (1), 165 (3), 151 (4), 137 (3), 135 (4), 123 (5), 121 (6), 109 (14), 98 (16), 96 (16), 95 (33), 93 (15), 82 (34), 81 (67), 79 (35), 68 (73), 67 (100), 55 (46), 54 (65), 53 (14), 43 (11), 41 (56), 39 (20), 29 (12), 27 (9). All spectral data are in accordance with reported data in the literature.^4^

**References to Experimental**

1. Csuk R, Niesen A, Tschuch G and Moritz G, *Tetrahedron* **60**: 6001–6004 (2004)

2. Cahiez G, Hablak V and Gager O, *Org Lett* **10**: 2389–2392 (2008)

3. Ando T, Ogura Y and Uchiyama M, *Agric Biol Chem* **52**: 1415–1423 (1988)

4. Baba A, Miyake Y and Kinsho T, Pub. No. US 2017/0297987 A1 (2017)
